# Generation of sas-6::ha by CRISPR/Cas9 editing

**DOI:** 10.17912/micropub.biology.000141

**Published:** 2019-08-01

**Authors:** Mary Bergwell, Amy Smith, Holly Lakin, Rebecca Slay, Jyoti Iyer

**Affiliations:** 1 University of Tulsa, 800 S. Tucker Dr, Tulsa, OK- 74104

**Figure 1. Generation of IYR001 strain containing sas-6(luv1[sas-6::ha]), and its localization in the early C. elegans embryo:**
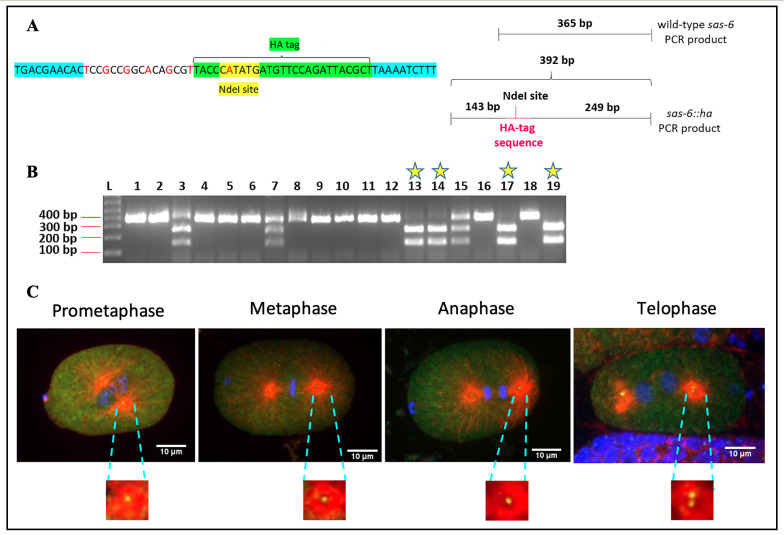
A) Left panel: Cartoon depicting nucleotide sequences flanking the *ha-tag* sequence in the designed repair template to generate the *sas-6::ha* Red text- Silent mutations introduced in the repair template; Teal highlight- part of homology arms. Right panel: Cartoon depicting DNA fragment sizes expected upon PCR followed by NdeI digestion for wild-type and *sas-6::ha* edited worms. B) Agarose gel electrophoresis of NdeI digested *sas-6* PCR products to screen for homozygous edited *sas-6(luv1[sas-6::ha])* worms. L: 1Kb DNA ladder, Yellow stars: *sas-6::ha* homozygous worms. C) Immunostaining with an anti-HA antibody shows localization of SAS-6::HA to the centrosomes (insets) and in the cytoplasm in 1-cell *C. elegans* embryos during different stages of mitosis. Microtubules (red), SAS-6::HA (green) and DNA (blue).

## Description

Centrosomes are comprised of a pair of barrel-shaped centrioles that are oriented at right angles to each other, and embedded in electron dense pericentriolar material. The centrosomes mediate spindle assembly and function as basal bodies to promote cilia and flagella formation (reviewed in Pintard and Bowerman, 2019 and Fırat-Karalar and Stearns, 2014). Six core proteins that are required for centriole duplication have been identified in *C. elegans.* These include the proteins SAS-7, SPD-2, ZYG-1, SAS-6, SAS-5 and SAS-4 (reviewed in Schwarz *et al*., 2018). The protein SAS-6 is frequently used to mark *C. elegans* centrosomes as it has been shown to be stably associated with *C. elegans* centrioles (Dammermann *et al*., 2004, Liedel *et al.*, 2005, Dammermann *et al*., 2008 and Balestra *et al.*, 2015). Previous efforts to tag the *sas-6* gene with fluorescent tags such as green fluorescent protein (GFP) have, however, resulted in considerable embryonic lethality (Dammermann *et al.*, 2008). One reason for this could be that large tags like GFP interfere with the function of the SAS-6 protein, thereby impairing its activity. Tagging *sas-6* with small epitope tags like HA could allow for visualization of endogenous SAS-6 localization without significantly impairing its activity. Although raising antibodies against endogenous *C. elegans* SAS-6 protein is an attractive alternative to epitope tagging, this is an expensive and time-consuming endeavor. Further, the specificity of antibodies that are raised in this manner cannot be guaranteed. On the other hand, antibodies against short epitope tags such as HA are commercially available, have been well-characterized and are available in a variety of different species (e.g. mouse, rabbit, goat, guinea pig, sheep, etc.) In this study, we have generated a worm strain (IYR001) with the endogenous *sas-6* gene *ha*-tagged using CRISPR/Cas9 editing. Specifically, we have inserted the coding sequence for the HA-tag (9 amino acids YPYDVPDYA) at the C-terminus of the SAS-6 protein. In this CRISPR experiment, we introduced a restriction site for the enzyme NdeI by silent mutation of the HA-tag in our repair template (Figure 1A, left panel). Therefore, all worms that exhibit the incorporation of the NdeI restriction site at the end of the *sas-6* gene must have a good chance of being successfully edited to incorporate our supplied repair template. The screening strategy for this CRISPR experiment is depicted in Figure 1A, right panel. Upon performing *sas-6* PCRs and digesting these PCR products with NdeI followed by agarose gel electrophoresis, we would expect to detect a single band of about 365 base pairs for wild-type, unedited worms, three bands of approximately 365 base pairs, 249 base pairs and 143 base pairs for heterozygous edited worms and two bands of about 249 base pairs and 143 base pairs respectively for homozygous edited worms (Figure 1A, right panel). As shown in Figure 1B, out of the 19 progeny of positive heterozygotes whose genotypes were analyzed, worm numbers 13, 14, 17 and 19 showed the presence of the homozygous *ha-*tagedit. We have also confirmed this CRISPR edit by DNA sequencing. At 20°C, *C. elegans* that are homozygous for the *sas-6::ha* edit have a slightly reduced average brood size of 240 (n=10) as compared with wild-type *C. elegans* that have an average brood size of 310 (n=12). However, importantly, *sas-6::ha* homozygotes do not exhibit any significant embryonic lethality (100% viable (n=10)) as compared with wild-type worms (99% viable (n=12)). We have performed immunostaining on the IYR001 strain with a monoclonal anti-HA antibody to determine the localization of SAS-6::HA in *C. elegans* embryos. SAS-6::HA displays a stereotypical centrosomal and cytoplasmic localization in 1-cell *C. elegans* embryos (Figure 1C). We believe that this strain will be a useful tool for *C. elegans* researchers studying SAS-6 localization and centrosome biogenesis.

## Reagents

**Immunostaining:** Immunostaining of *C. elegans* embryos was performed as described previously (O’Connell and Golden, 2014) except that TBSBT (TBST with BSA) was used for the blocking and washing steps instead of PBSBT (PBST with BSA). The anti-HA antibody was used at a 1:1000 dilution [Cell Signaling (Catalog # 3724S)] and the anti-tubulin antibody (DM1a) was used at a 1:50 dilution [Santacruz Biotechnology (Catalog # sc-32293)]. Anti-rabbit Alexa fluor 488 (Catalog # A-11034) and anti-mouse Alexa fluor 568 (Catalog # A-11004) secondary antibodies were purchased from Thermo Fisher Scientific and used at a 1:1000 dilution. The embryos were mounted in vectashield containing DAPI [Vector laboratories (Catalog # H-1200)].

**CRISPR/Cas9 editing protocol:** CRISPR/Cas9 editing was performed as described previously (Paix *et al*., 2015). The progeny of successfully injected worms were subjected to PCR [MyTaq Redmix, Bioline] followed by restriction digestion with NdeI [New England Biolabs]. Positive edits were identified by agarose gel electrophoresis of digests.

**Sequences used for CRISPR/Cas9 editing:**The tracrRNAsequence*, dpy-10* crRNA and *dpy-10* repair template sequences have been described in Paix *et al*., 2015.*sas-6* crRNA sequence: 5′ – ATTTTATCGTTGAGCGGGTG – 3′*sas-6::ha* repair template sequence:

5'- TATTTTCAAGTAAAGGACAAGAAAAAATCAATAAAAAAGATTTTAAGCGT
    AATCTGGAACATCATATGGGTAACGCTGTGCCGGCGGAGTGTTCGTCACA
    CTTGAACCAGTAGTCTCGTCGGCGATTAGTTGA – 3'

SAS-6::HA sequencing forward primer: 5′ – CCCCATTCCGTGACAATACA – 3′SAS-6::HA sequencing reverse primer: 5′ – CCTTACCTCTTGAACTGCC – 3′Strain name: IYR001 Allele name: *sas-6(luv1[sas-6::ha])*The IYR001 strain will be made available upon request.

**Imaging:** The immunostained embryos were viewed using an Olympus IX83 Yokagawa CSU-X1 spinning disk confocal microscope and images were captured with a prime 95B CMOS camera.
